# Resolvin D5 (RvD5) Reduces Renal Damage Caused by LPS Endotoxemia in Female Mice

**DOI:** 10.3390/molecules28010121

**Published:** 2022-12-23

**Authors:** Renato D. R. Cardoso, Sandmary D. Chambo, Tiago H. Zaninelli, Beatriz H. S. Bianchini, Matheus Deroco Veloso da Silva, Mariana M. Bertozzi, Telma Saraiva-Santos, Anelise Franciosi, Geovana Martelossi-Cebinelli, Pamela E. Garcia-Miguel, Sergio M. Borghi, Rubia Casagrande, Waldiceu A. Verri

**Affiliations:** 1 Laboratory of Pain, Inflammation, Neuropathy and Cancer, Department of Pathology, Centre of Biological Sciences, Londrina State University, Londrina 86057-970, Brazil; 2 Department of Pharmaceutical Sciences, Centre of Health Science, Londrina State University, Londrina 86039-440, Brazil

**Keywords:** resolvin D5, lipopolysaccharide, endotoxemia, inflammation

## Abstract

In self-revolving gram-negative *Escherichia coli* infection, Resolvin D5 (RvD5) was found to enhance bacteria phagocytosis and reduce the production of inflammatory mediators, contributing to the resolution of infection. LPS (lipopolysaccharide) is a gram-negative bacterial structure product which activates the immune system and, at high doses, leads to endotoxemia. To our knowledge, the effect of RvD5 against LPS endotoxemia has not been investigated to date. Female Swiss mice received an i.p. treatment with RvD5 (0.1, 1 or 10 ng/animal). After 1 h, they were stimulated with LPS (10 mg/kg, i.v.), and samples were collected after additional 6 h. The resulting data demonstrated that RvD5 protected the kidneys (urea and creatinine serum levels) from tissue injury. These effects were related to an improvement in histopathological parameters and a reduction of enzymatic markers of leukocyte infiltration, pro-inflammatory cytokine (IL-1β, TNF-α, and IL-6) production, and oxidative stress. Antioxidant markers were also increased by RvD5, but IL-10 (an anti-inflammatory cytokine) levels were unaltered. We also observed that RvD5 reduced the infiltration of CD45^+^ hematopoietic cells into the kidneys, reduced the activation of NFκB and promoted the Nrf2 pathway by reducing Keap-1 levels. Our data indicate that RvD5 may be a therapeutic possibility to reduce kidney lesions in LPS endotoxemia.

## 1. Introduction

Sepsis is a major disease in health care, since approximately 50% of affected patients do not survive [1,2]. Survival rates can be even lower, depending on the country [3]. The condition induces multiorgan failure [4,5]. For instance, acute kidney injury is a major problem in sepsis because the risk of renal failure is high even if the patient survives [6,7,8]. This loss of kidney function has long term consequences to the patient and health care systems [6,9]. Lipopolysaccharides (LPS) of gram-negative bacteria can mimic some of the effects of sepsis. More specifically, a high systemic dose of LPS induces endotoxemia, which differs from sepsis by the lack of a viable proliferating microorganism, making it possible to focus on endotoxemia and not bacterial proliferation [10,11]. In fact, LPS endotoxemia also induces acute kidney injury via toll-like receptor 4 (TLR-4) activation, NFkB activation, oxidative stress, and cytokine production [12,13].

Resolvins are SPMs derived from the omega-3 fatty acids EPA (Eicosapentaenoic acid) and DHA (Docosapentaenoic acid), which are primary substrates of these biomolecules. Resolvins can be subdivided according to their precursor molecule into Resolvins of the E (RvE) series for those derived from EPA, and Resolvins of the D (RvD) for those derived from DHA [14]. DHA-derived RvDs are synthesized by different cells (polymorphonuclear cells and macrophages) through the action of 15-lipoxygenase (15-LOX), which transforms DHA into 17S-hydroperoxy-DHA. This intermediate, in turn, undergoes the action of 5-lipoxygenase (5-LOX), generating six RvD, which differ structurally according to their position, chirality and the number of hydroxyl residues [14,15,16], including the RvD5.

Resolvin D5 (RvD5) or 7(*S*),17(*S*)-diHDHA has been identified in the inflammatory milieu of *Escherichia coli* infection in a bacterial load that produces self-resolving inflammation. Interestingly, RvD5 was shown to enhance bacteria phagocytosis, which is an important function to promote the killing of bacteria. RvD5 also reduced the production of pro-inflammatory cytokines, contributing to the reduction of inflammation [17]. Additionally, RvD5 was shown to protect against *Citrobacter rodentium* infection of infant mouse as well as reinfection. Normally, this infection would be characterized by high *C. rodentium* load that increases rapidly and remains sustained, resulting in intestinal inflammation with necrosis and reaching the blood stream; however, RVD5 reversed this scenario [18]. Thus, RvD5 seems to protect from some bacterial infections.

Even without infection, RvD5 is also active. It reduces anxiety and depressive like behaviors by down-modulating IL-1β production in the hippocampus and prefrontal cortex of diabetic mice [19]. It was also shown to reduce joint inflammation in a mouse model of zymosan arthritis. This activity involved the inhibition of CD4^+^ T cell proliferation and Th17 cell differentiation, together with an increase of regulatory T cell differentiation and a reduction of osteoclast differentiation and osteoclastogenesis [20]. Thus, RvD5 also reduces adaptive immunity-dependent inflammation. Interestingly, the analgesic effect of RvD5 in a chemotherapy model triggered neuropathy in male but not in female rodents [21], which raises the possibility that this lipid may not be active in females.

Thus, RvD5 can reduce infection, including systemic bacterial infection [17,18]; however, its activity in LPS endotoxemia has not been explored so far. This is important, because such knowledge may help to ascertain whether the activity of RvD5 is related solely to proliferating bacteria or if this lipid can inhibit the systemic inflammatory response and organ failure caused by bacterial products such as LPS. Therefore, the present study investigated the activity of RvD5 against LPS endotoxemia-induced kidney injury. We focused on LPS endotoxemia-induced acute kidney injury because of the long-term consequences of this type of organ damage to the patient and health care systems. Finally, our study was initiated with female mice to check if RvD5 would be inactive. If this was the case, we would then move to male mice. However, as we will show, RvD5 reduced LPS endotoxemia acute kidney injury in female Swiss mice.

## 2. Results

### 2.1. RvD5 Decreases the Levels of Renal Injury Markers in Plasma

**Figure 1:**
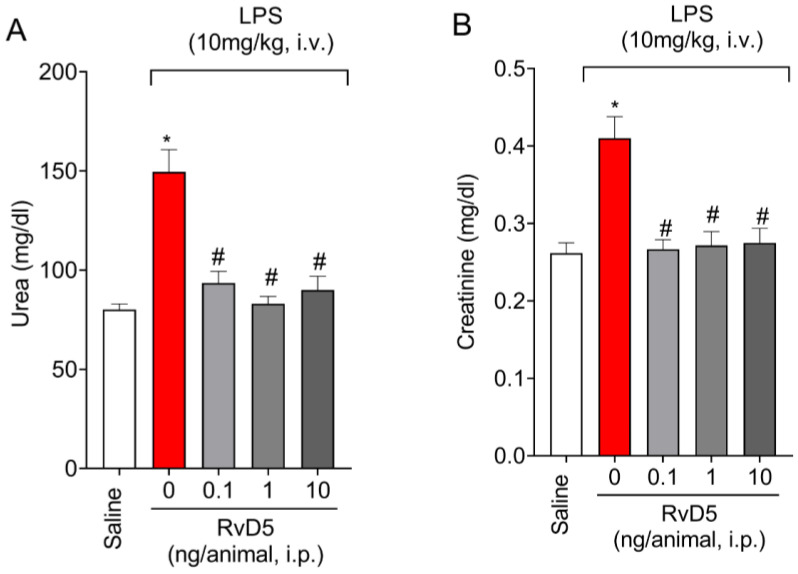
RvD5 decreases endotoxemia-triggered renal injury. The plasma levels of urea (**A**) and creatinine (**B**) were quantitated. Results are presented as mean ± SEM (standard error of the mean) of six animals per group and are representative of two replicates. (* *p* < 0.05 vs. saline, # *p* < 0.05 vs. LPS 10 mg/kg; one-way ANOVA followed by Tukey’s post-test).

We started by performing a dose-response curve to evaluate the activity of RvD5 (0.1; 1 or 10 ng/animal, i.p.). We used a pharmacological approach to test RvD5 as a drug; however, the selected dose range was based on literature data that was obtained using up to 10 ng of RvD5 with positive activities [19], as well as data on cellular and tissue production of RvD5, detecting levels ranging from approximately 100 pg and 10 ng [22,23,24]. Blood levels of urea and creatinine, which are clinical markers of kidney injury, were measured 6 h after LPS stimulation in plasma samples. The systemic administration of LPS (10 mg/kg, i.v.) significantly increased plasma levels of urea (Figure 1A) and creatinine (Figure 1B). Treatment with RvD5 significantly decreased the plasma levels of those markers (Figure 1A,B). Therefore, RvD5 was shown to protect kidney from endotoxemia-triggered lesions.

### 2.2. RvD5 Decreases MPO and NAG Activities in the Kidneys

**Figure 2:**
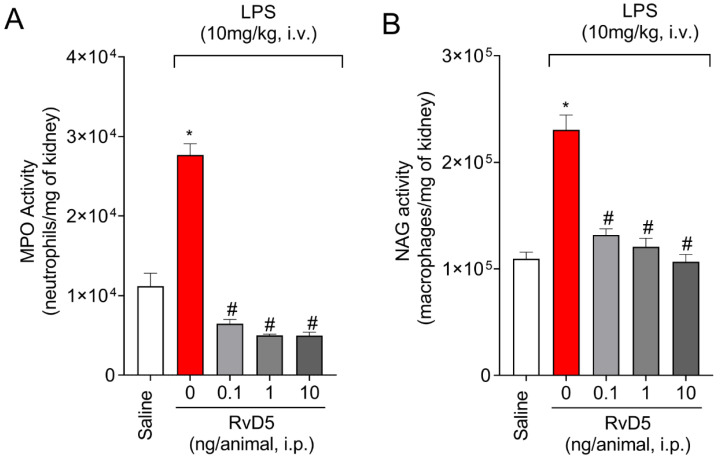
RvD5 decreased MPO and NAG activities in the kidneys. MPO (**A**) and NAG (**B**) activities were determined in kidney samples. Results are presented as mean ± SEM of six animals per group and are representative of two replicates. (* *p* < 0.05 vs. saline, # *p* < 0.05 vs. LPS 10 mg/kg; one-way ANOVA followed by Tukey’s post-test.

Leukocytes activation and recruitment to affected tissue are crucial points in the inflammatory process, as well as in inflammatory tissue injury. We determined the levels of MPO and NAG activities in the kidneys. We observed a significant increase in MPO and NAG activities after systemic administration of LPS (Figure 2). All tested doses decreased MPO and NAG activities in the kidneys (Figure 2A,B).

### 2.3. RvD5 Decreases the Production of Pro-Inflammatory Cytokines but Does Not Affect Anti-Inflammatory Cytokine Levels in The Kidneys

**Figure 3:**
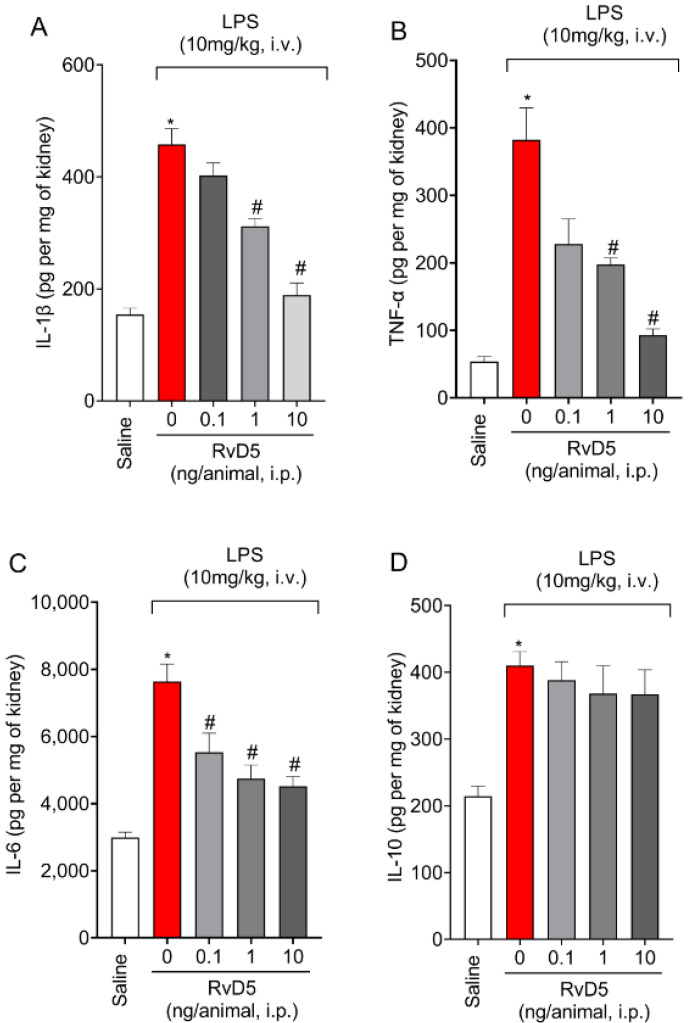
RvD5 reduced LPS endotoxemia-triggered production of IL-1β, TNF-α and IL-6 without affecting IL-10 levels in the kidneys. IL-1β (**A**), TNF-α (**B**), IL-6 (**C**) and IL-10 (**D**) levels were determined in kidney homogenates. Results are presented as mean ± SEM of six animals per group and are representative of two replicates. (* *p* < 0.05 vs. saline, # *p* < 0.05 vs. LPS 10 mg/kg; one-way ANOVA followed by Tukey’s post-test.

LPS endotoxemia induced an increase of IL-1β (Figure 3A), TNF-α (Figure 3B) and IL-6 (Figure 3C) levels in the kidneys (Figure 3). The levels of all three cytokines were decrease by RvD5 treatment at doses of 1 and 10 ng/animal (i.p.) (Figure 3). The lowest tested dose of RvD5 also significantly reduced the levels of IL-6 (Figure 3C) but had no significant effect on the production of IL-1β (Figure 3A) or TNF-α (Figure 3B). LPS endotoxemia also increased the levels of IL-10 in the kidneys (Figure 3D). IL-10 has, in general, an anti-inflammatory activity and is co-released with pro-inflammatory cytokines. Thus, the resultant inflammation depends on the balance between pro- and anti-inflammatory cytokines. Some drugs may act by increasing IL-10 levels beyond regular production, which results in a reduction of inflammation [22,23,24]. RvD5 did not alter IL-10 production in the kidneys caused by LPS endotoxemia (Figure 3D), which suggests that this lipid was not acting by, for instance, up-regulating IL-10 production.

### 2.4. RvD5 Reestablishes Antioxidant Capacity

**Figure 4:**
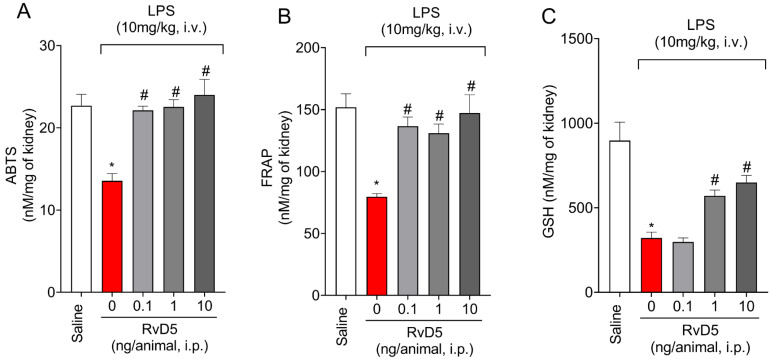
RvD5 reestablishes tissue antioxidant capacity. ABTS (**A**), FRAP (**B**) and GSH (**C**) were quantitated in the kidneys. Results are presented as mean ± SEM of sixanimals per group and are representative of two replicates. (* *p* < 0.05 vs. saline, # *p* < 0.05 vs. LPS 10 mg/kg; one-way ANOVA followed by Tukey’s post-test).

The ABTS (2,2′-azinobis-(3-ethylbenzothiazoline-6-sulfonic acid)) and the FRAP (Ferric Reducing Antioxidant Power) assays are methods to evaluate the total antioxidant capacity of a sample. Systemic administration of LPS decreased antioxidant capacity as per ABTS (Figure 4A) and FRAP (Figure 4B) methods and decreased GSH levels (Figure 4C) in the kidneys. Additionally, RvD5 reestablished the total antioxidant capacity at all tested doses of RvD5 as per ABTS (Figure 4A) and FRAP (Figure 4B) and methods. A dose of 0.1 ng of RvD5 did not alter the reduction of GSH, contrasting with the results of doses of 1 and 10 ng of RvD5, which had a significant effect against GSH reduction in the kidneys by LPS endotoxemia (Figure 4C).

### 2.5. RvD5 Decreases Oxidative Stress

**Figure 5:**
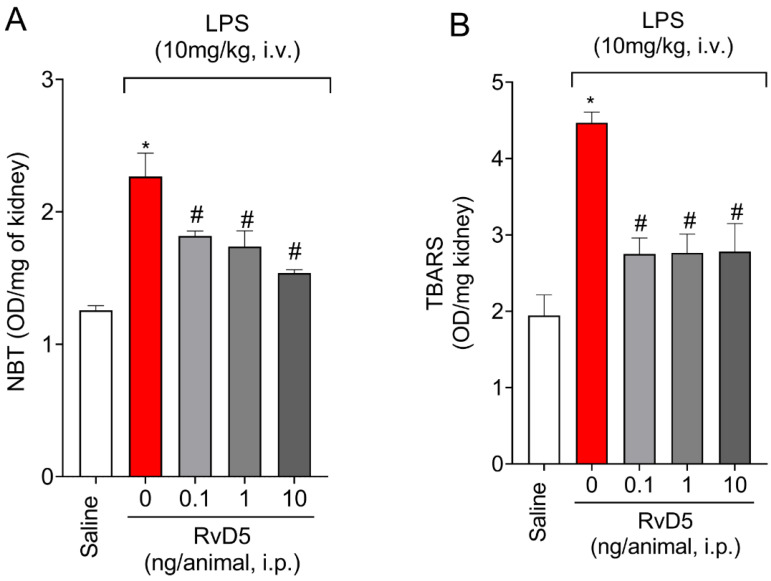
RvD5 reduces superoxide anion production and lipid peroxidation levels in the kidneys. Superoxide anion production by the NBT method (**A**) and lipid peroxidation by the TBARS method (**B**) were quantitated in the kidneys. Results are presented as standard ± SEM (standard error mean) of six animals per group and are representative of two replicates. (* *p* < 0.05 vs. saline, # *p* < 0.05 vs. LPS 10 mg/kg; one-way ANOVA followed by Tukey’s post-test).

Superoxide anion production (NBT) and lipid peroxidation levels (TBARS) increased in the kidneys upon LPS endotoxemia (Figure 5). RvD5, at all doses tested, decreased superoxide anion production (Figure 5A) and TBARS levels (Figure 5B) in the kidneys.

### 2.6. RvD5 Decreases Histopathological Changes, Leukocyte Infiltration, and Necrosis in the Kidneys

**Figure 6:**
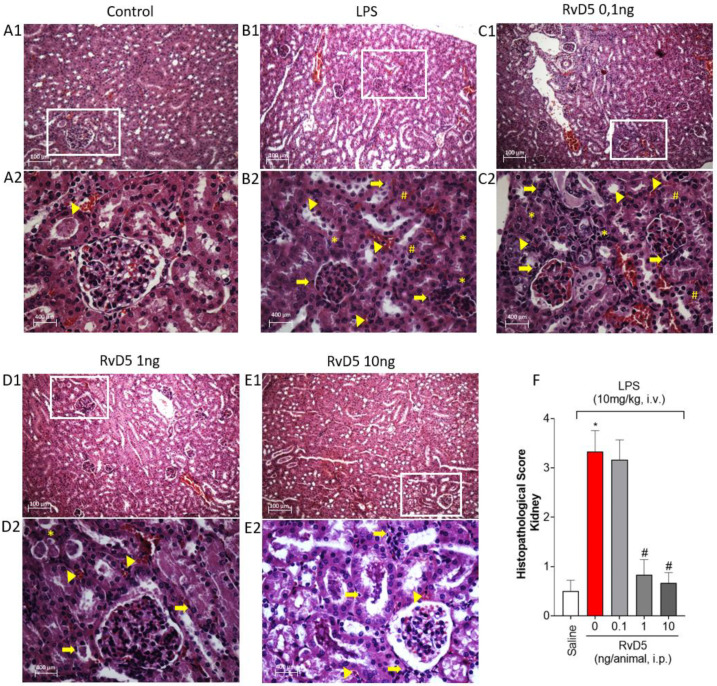
RvD5 preserves cortical kidney architecture in LPS endotoxemia. Representative photomicrographs of renal histology (hematoxylin and eosin, magnification 10× and 40×). Control (**A1**), LPS + vehicle (**B1**) (10 mg/kg, i.v.), RvD5 treated at 0,1 ng (**C1**), 1 ng (**D1**) and 10 ng (**E1**). (**A2**–**E2**) represent the fields with the zooms highlighted by the white rectangular inserts. Arrow: inflammatory infiltrate; Arrowhead: red blood cells; * necrotic changes; # bleeding focus. (**F**) Histopathological score. Results are presented as median + interquartile range analyzed by Kruskal-Wallis followed by Dunn’s test of six animals per group and are representative of two replicates. Scale bars: 100 and 400 µm. (* *p* < 0.05 vs. saline, # *p* < 0.05 vs. LPS 10 mg/kg).

Kidneys samples were processed and stained with H&E. Compared to the negative control group (Figure 6(A1,A2)), LPS endotoxemia caused moderate hydropic degeneration, mild mixed inflammatory cells (<25 cells), mild necrotic involvement (up to 25%) and presence of blood cells in the parenchyma of the kidneys (Figure 6(B1,B2)). The effect of the lowest dose of RvD5 (Figure 6(C1,C2)) did not differ from the group stimulated with LPS and treated with vehicle (Figure 6(B1,B2)). On the other hand, treatment with doses of 1 and 10 ng (Figure 6(D1–E2)) decreased the number of inflammatory cells, with absence of necrotic signs and maintenance of hydropic degeneration. These results were plotted as a score, as presented in Figure 6F.

### 2.7. RvD5 Reduces the Infiltration of CD45^+^ Hematopoietic Leukocytes, Pnfkb and Keap-1 Staining in the Kidneys of Endotoxemic Mice

**Figure 7:**
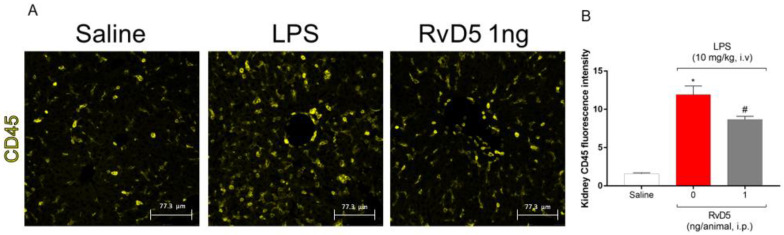
RvD5 reduces the infiltration of CD45+ hematopoietic leukocytes staining in the kidneys. Representative images (**A**) and CD45^+^ fluorescence intensity (**B**). Results are presented as standard ± SEM (standard error mean) one-way ANOVA followed by Tukey’s post-test) of six animals per group and are representative of two replicates. Scale bar: 77.3 µm. (* *p* < 0.05 vs. saline, # *p* < 0.05 vs. LPS 10 mg/kg).

We further investigated the underlying mechanisms of RvD5 reduction of LPS endotoxemia-triggered kidney injury. We selected the dose of 1 ng of RvD5, since it achieved the same activity as the dose of 10 ng, while a dose of 0.1 ng did not reduce all parameters altered by LPS. Endotoxemia caused an accumulation of CD45^+^ hematopoietic cells in the renal cortex, as determined by fluorescence intensity (Figure 7A,B), which agrees with the MPO and NAG activities data (Figure 2) and histopathological evaluation (Figure 6).

**Figure 8:**
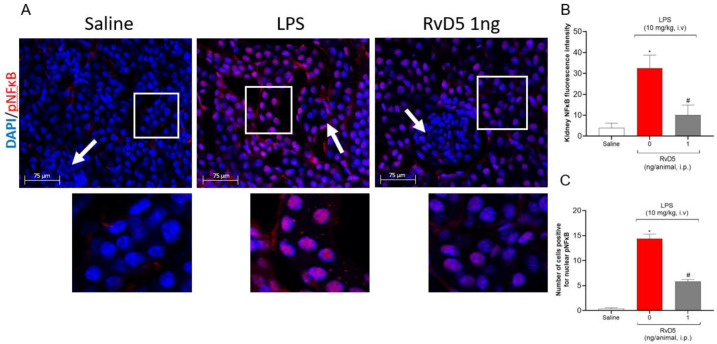
RvD5 reduces pNFκB staining in the cortical region of the kidneys. Representative images (**A**), pNFκB fluorescence intensity (**B**), and number of cells positive for nuclear pNFκB (**C**). White squares in reduced magnification imagens (upper panels) indicate the region of the zoomed images (lower panels) where the presence of phosphorylate (red; pNFκB) in the cell nucleus (blue; DAPI) is shown. Glomeruli are indicated in cortical regions by white arrows. Results are presented as standard ± SEM (standard error mean) one-way ANOVA followed by Tukey’s post-test) of six animals per group and are representative of two replicates. Scale bar: 75 µm. (* *p* < 0.05 vs. saline, # *p* < 0.05 vs. LPS 10 mg/kg).

As we observed an increase of pro-inflammatory cytokine production and oxidative stress, we proceeded with the staining of two redox sensitive pathways, i.e., the NFκB and the Keap-1/Nrf2, in the renal cortex of the samples [25,26,27]. Endotoxemia increased the pNFκB co-localization with DAPI, indicating a nuclear localization of pNFκB and its activation, which was reduced by RvD5 (Figure 8A–C).

**Figure 9:**
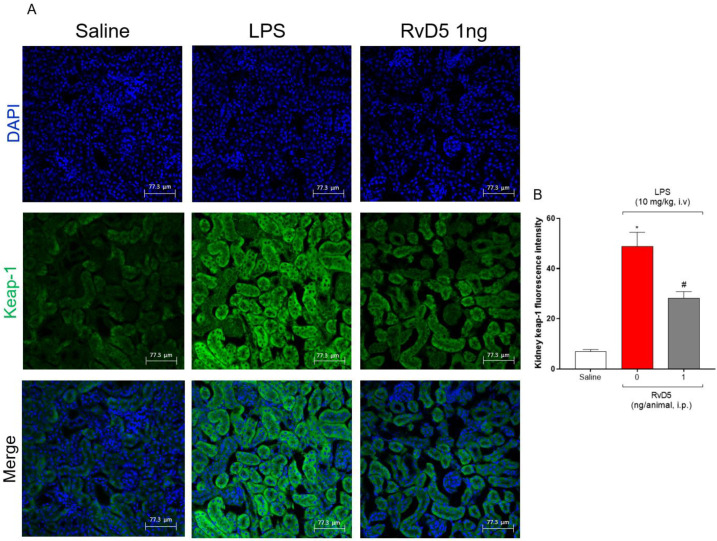
RvD5 reduces Keap-1 staining in the kidneys. Representative images (**A**) and Keap-1 fluorescence intensity (**B**). Results are presented as standard ± SEM (standard error mean) one-way ANOVA followed by Tukey’s post-test) of six animals per group and are representative of two replicates. Scale bar: 77.3 µm. (* *p* < 0.05 vs. saline, # *p* < 0.05 vs. LPS 10 mg/kg).

Keap-1 maintains the transcription factor Nrf2 in an inactive state and induces its ubiquitination and proteasomal degradation. Thus, elevated Keap-1 staining means a reduction of Nrf2 transcription activity and, consequently, diminished antioxidant response [26,28,29]. LPS endotoxemia increased Keap-1 staining, which was reduced by RvD5 (Figure 9A,B). Thus, RvD5 reduces the infiltration of CD45^+^ hematopoietic cells, as well as reducing NFκB activation. Additionally, it likely facilitates Nrf2 activation by reducing Keap-1 in the kidneys of LPS endotoxemic female mice.

## 3. Discussion

This study analyzed the activity of RvD5 in an LPS-induced endotoxemia model in female mice. We demonstrated that the administration of LPS triggers kidneys injury. This effect was confirmed by plasma markers, renal tissue markers, histopathological changes and immunofluorescence staining of kidney samples. LPS endotoxemia increased the plasma levels of urea and creatinine. In the kidneys, LPS endotoxemia increased MPO and NAG enzymatic activities, pro-inflammatory cytokine production and histopathological changes compatible with inflammatory damage. LPS also decreased antioxidant defenses in the kidneys. In agreement with those data, LPS endotoxemia enhanced oxidative stress markers, infiltration of CD45^+^ hematopoietic cells in the kidneys, NFκB activation and Keap-1. RvD5 reversed all these changes, which collectively explain the beneficial effect of this lipid against LPS endotoxemia-triggered renal damage. To our knowledge, this has not been previously demonstrated.

Endotoxemia models are used to simulate an acute inflammatory response with potential evolution to sepsis [10]. This approach is used to isolate the inflammatory response from the microbicidal events that occur during sepsis. In a pre-clinical scenario, LPS is the most widely used molecule to establish the endotoxemia inflammatory process [10,30]. LPS administration, either intravenously or intraperitoneally, induces systemic inflammation, resulting in immune cell activation and the release of inflammatory mediators, with consequent vascular/hemodynamic changes and the generation of reactive oxygen and nitrogen species, leading to organ and tissue dysfunction, followed by organ function failure [10,31,32]. RvD5 stimulates important activities such as phagocytosis of *E. coli* [17] and reduces lethal infection with *C. rodentium* in infant mouse. These are gram-negative bacteria that have LPS as an important virulence factor [18]. Thus, investigation of the activity of RvD5 in LPS endotoxemia was crucial, since it provides information about the activity of this lipid against inflammatory organ damage without ongoing microorganism proliferation.

The kidneys are affected in LPS endotoxemia, which can escalate to acute renal failure with progressive decrease of glomerular filtration rate, and finally, chronic kidney disease, if the patient survives [33,34]. In fact, inadequate treatment of acute kidney injury can accelerate, progressing to chronic kidney disease [35]. In models of kidney injury in endotoxemia, swelling and tubular degeneration have been demonstrated, with an increase in the expression of pro-inflammatory cytokines, inflammatory cell influx, and high serum levels of urea, creatinine, and free radicals [36,37]. In addition, LPS increases the expression of high mobility group box 1 (HMGB1), inducible nitric oxide synthase (iNOS), B-cell lymphoma 2 (Bcl-2), Bcl-2 associated X (BAX), caspase-3, and phosphorylated forms of NFκB (p65) and mitogen-activated protein kinases (MAPK) [38]. In the present study, we demonstrated that RvD5 also protects the kidneys, as observed by the improvement of plasma urea and creatinine levels. These are the most common plasma markers to evaluate renal function [39,40].

RvD5 reduced the leukocyte counts in the kidneys, as observed by MPO and NAG enzymatic activities, but also through a histopathological examination and CD45^+^ hematopoietic cells staining in LPS endotoxemia. Infiltrating leukocytes contribute, for instance, to the production of pro-inflammatory cytokines. We quantitated IL-1β, TNF-α, and IL-6 as examples of cytokines which are widely accepted as pro-inflammatory ones in many diseases, including endotoxemia [41]. RvD5 treatment of THP-1 cells reduces LPS-induced production of IL-6 and CC motif chemokine ligand 5 (CCL5) through the modulation of MAPK and NFκB, demonstrating the mechanisms by which RvD5 can achieve anti-inflammatory activity in vitro [42]. However, whether RvD5 was active in vivo in reducing cytokine production initiated by LPS was unknown. IL-10 is an anti-inflammatory cytokine that is co-released with pro-inflammatory cytokines. The intensity of inflammation is a result of the balance between anti-inflammatory and pro-inflammatory molecules. In this sense, we observed that LPS induced the production of IL-10. Drugs of varied classes can reduce inflammation by increasing IL-10 levels [43,44,45,46]. In turn, IL-10 acts, for instance, by reducing the production of inflammatory cytokines such as TNF-α and IL-1β [22,47]. However, RvD5 did not act by a mechanism that was dependent on increasing IL-10 production. Recruited leukocytes also produce reactive oxygen species (ROS). MPO itself is an enzyme that uses hydrogen peroxide to produce hypochlorite, which is a toxic molecule that is capable of activating proteases and contributing to tissue damage [48,49]. RvD5 could reestablish oxidative balance in the kidneys since it was shown to reduce LPS endotoxemia oxidative stress (down-modulated endogenous antioxidants and increased oxidative stress markers). ROS play a dual role, since they are capable of inducing tissue injury [50,51] but also of signaling molecules regulating, for instance, neutrophil recruitment [52,53]. Therefore, this scenario, in which RvD5 reduces LPS endotoxemia recruitment of leukocytes, cytokine production, and oxidative stress, is intimately related to a reduction of renal injury.

NFκB and Keap-1/nuclear factor erythroid 2–related factor 2 (Nrf2) are two transcription factor pathways that are sensitive to oxidative stress. The difference is that NFκB upregulates inflammation while Nrf2 increases antioxidant and anti-inflammatory responses [54,55]. The co-localization of pNFκB within the nucleus of cortex kidney cells was increased by LPS endotoxemia and reduced by RvD5. This mechanism of action of RvD5 explains the overall reduction of inflammation, leukocyte recruitment, cytokine production, and oxidative stress. Keap-1 maintains Nrf2 in an inactive state in the cell cytoplasm, rendering it susceptible to ubiquitination and proteasomal degradation. Therefore, the increase of Keap-1 staining indicates a reduction of Nrf2 activity, while conversely, a reduction of Keap-1 staining is indicative of enhanced Nrf2 activity [25,26,27,28,29]. RvD5 reduced the Keap-1 staining, indicating an enhancement of Nrf2 activity. In fact, RvD5 inhibited the LPS endotoxemia reduction of GSH levels. Since GSH is a downstream target of Nrf2 [27], the Keap-1 and GSH data are aligned, indicating an increase of Nrf2 activity.

RvD5 has an analgesic effect in chemotherapy-induced neuropathy and formalin nociception. This analgesic activity occurred in male, but not in female mice [21]. Taking this information into account, we reason that the first step was to start investigating the activity of RvD5 in female mice, and only move to male mice if this lipid was inactive in females. However, RvD5 was shown to be active in reducing LPS endotoxemia in female mice. This led us to speculate that potential sex dimorphism in the activity of RvD5 would be dependent on nociceptor sensory neurons rather than on inflammation processes such as that triggered by LPS endotoxemia. The high intensity of inflammation of endotoxemia compared to other titrated stimuli in doses that do not robustly activate the entire organism may help to explain the observed sex differences. The authors of [21] used CD1 and C57BL/6 mice, while we used Swiss mice; thus, this difference in mouse strains may help to explain the observed sex dimorphism. These results also indicate that whether RvD5 is active in females or not should be tested in each condition until we have a better knowledge base with which to ascertain the diseases in which this SPM might be useful, as well as a better understanding of potential sex dimorphisms, before entering clinical studies.

## 4. Materials and Methods

### 4.1. Animals

The present study was conducted with pathogen-free female Swiss mice (8-weeks old, weighing between 20–25 g). Animals were provided by Universidade Estadual de Londrina animal facility (Londrina, PR, Brazil) and were housed in a climatized room (21 ± 1 °C) with free access to water and food, and a light/dark cycle (12/12 h). The animals were kept into standard polypropylene cages measuring 41 × 34 × 16 cm (Insight^®^ Equipamentos, Ribeirão Preto, Brazil) with a maximum of 10 animals per cage. All procedures were performed in accordance with local, national, and international standards of ethics. Animal handling and welfare were approved by the Universidade Estadual de Londrina Ethics Committee for animal experimentation (CEUA-UEL, protocol number 052.2021). Animal studies are reported in compliance with the ARRIVE guidelines [56]. The total number of animals used in the present study was 156.

### 4.2. Experimental Procedures and Endotoxemia Model

RvD5 (7S,17S-dihydroxy-4Z,8E,10Z,13Z,15E,19Z-docosahexaenoic acid), Cayman Chemical, Ann Arbor, MI, USA, 0,1; 1 and 10 ng/animal) or vehicle (0.1% ethanol in saline) were administrated intraperitoneally (i.p.). After 60 min, mice received LPS (10 mg/kg, from *E. coli*—Santa Cruz Biotechnology) or vehicle (0.9% saline solution) intravenously (i.v.; caudal vein). Animals were divided into five equal sized groups consisting of six animals per group. Group 1: 0.1% ethanol in saline (i.p.) + saline (i.v.); Group 2: 0.1% ethanol in saline (i.p.) + LPS 10 mg/kg (diluted in saline, i.v.); Group 3: RvD5 0.1 ng/animal (diluted saline—i.p.) + LPS 10 mg/kg (i.v.); Group 4: RvD1 1 ng/animal (i.p.) + LPS 10 mg/kg (i.v.); and Group 5: RvD1 10 ng/animal (i.p.) + LPS 10 mg/kg (i.v.). For all assays, samples were collected 6 h after LPS administration.

### 4.3. Determination of Renal Injury Markers Levels in the Plasma

Blood was collected by cardiac puncture under isoflurane anesthesia (3% *v*/*v* in oxygen), followed by an increase in isoflurane concentration for euthanasia (5% *v*/*v* in oxygen). Blood samples were placed in microcubes containing 50 µL of porcine sodium heparin (5000 IU/mL, Blau Farmaceutica S.A., SC, Brazil) and centrifuged (3500 rpm × 10 min at 4 °C) for plasma separation. The determination of plasma urea levels was quantitated by the enzymatic method (Laborlab—São Paulo—Brazil), while creatinine levels were quantitated by the kinetic method (Gold Análise Diagnóstica—Minas Gerais—Brazil) using Selectra automated equipment. Results were expressed in mg/dL [43].

### 4.4. Myeloperoxidase (MPO) Activity Assay

MPO activity was used as an indirect measure of neutrophils in the kidneys. Samples were collected in 200 µL of K_2_HPO_4_ buffer solution (pH 6.0), containing 0.5% of HTAB, and were homogenized with a tissue homogenizer. Subsequently, samples were centrifuged (14,000 RPM × 4 °C × 2 min) and the supernatant was separated. The kidney homogenate supernatant was added to 200 µL of 50 mM phosphate buffer solution (pH 6.0), containing 0.167 mg/mL of *o*-dianisidine dihydrochloride and 0.015% of hydrogen peroxide. Absorbances were recorded with a microplate spectrophotometer (ThermoScientific, Multiskan GO, Vantaa, Finland) at 450 nm. MPO activity was expressed as myeloperoxidase activity (number of neutrophils × 10^3^/mg of tissue) compared to a neutrophil standard curve [57].

### 4.5. N-acetylglucosaminidase (NAG) Activity Assay

NAG activity was determined as described by Hohmann et al. [58]. Briefly, the enzyme activity was measured from the supernatant obtained in the MPO assay. Samples were placed in a microplate and 80 µL of 50 mM phosphate buffer (pH 6) was added. The reaction started after the addition of 2.24 mM of 4-nitrophenyl *N*-acetyl-β-d-glucaminide, followed by incubation at 37 °C for 10 min. Finally, 100 µL of 0.2M glycine buffer (pH 10.6) was added to the reaction. The enzyme activity was determined spectrophotometrically at 400 nm; the results were expressed as NAG activity (macrophages × 10^3^/mg tissue) compared to the macrophage standard curve.

### 4.6. Cytokine Measurement

Kidneys were collected in phosphate buffered saline for cytokines level determination. Samples were homogenized, centrifuged (3000 rpm × 4 °C × 15 min), and the supernatant was used to measure the levels of tumor necrosis factor-α (TNF)-α, interleukin (IL)-1β, IL-6, and IL-10 by enzyme-linked immunosorbent assay (ELISA). The assay was conducted following manufacturer’s instructions (BioLegend—San Diego, CA, USA).

### 4.7. ABTS and FRAP Assays

Kidney samples were collected in 500 µL of 1.15% KCl buffer, homogenized, and centrifuged (1500 rpm/4 °C/10 min). To measure the ability of each sample to resist oxidative stress, the modified ABTS (2,2-azinobis (3-ethylbenzothiazoline-6-sulfonic acid) method primarily described by Re et al. [59] was used. ABTS reagent was added to the supernatant and allowed to incubate for 6 min at room temperature, and the samples were read in a microplate spectrophotometer with at 730 nm. To evaluate the FRAP (ferric reducing ability of plasma), 150 µL of the FRAP reagent was added to the supernatant and read at 595 nm. The results were compared to a Trolox standard curve (30 mM, final concentration) and expressed as nM/Trolox equivalent/mg of tissue at both evaluations.

### 4.8. Determination of Thiobarbituric Acid Reactive Substances (TBARS) Levels

Free radicals, when in contact with lipids, destabilize these molecules and produce malondialdehyde (MDA). In turn, MDA amplifies the inflammatory process. Therefore, a TBARS test was used to detect the presence of MDA in samples using thiobarbituric acid (TBA). TBA reacts with MDA, forming a new compound which can be detected spectrophotometrically. Sample were homogenized in 500 µL of 1.15% KCl. Then, 50% trichloroacetic acid (TCA) was added, and after centrifugation (1000× *g*, 3 min, 4 °C), the supernatant was reacted with 50 µL of 1% TBA at 95 °C for 60 min or until the color changed to pink. Then, the absorbance was measured at 572 nm and 535 nm. The results were expressed as 572 nm–575 nm OD/mg of protein [43].

### 4.9. Nitroblue Tetrazolium (NBT) Assay

Quantification of superoxide anion production was performed using the NBT assay, as previously described and adapted to be performed in microplates [60]. Briefly, 50 µL of sample homogenate was used and incubated in microplates with 100 µL of solution containing the NBT reagent (1 mg/mL) for 1 h at room temperature and protected from direct light. After incubation, the entire contents of the microplate were removed and 120 µL of 2M KOH solution and 120 µL of pure DMSO were added. The relative production of superoxide anions was measured spectrophotometrically at a 600 nm. The results were expressed in OD/mg of protein.

### 4.10. Determination of Reduced Glutathione (GSH) Level

Kidneys were homogenized in 500 µL of 0.02M EDTA. Two hundred microliters of homogenate were added to 25 µL of 50% trichloroacetic acid, incubated for 15 min at room temperature, and centrifuged (4000 rpm × 4° × 15 min). For the assay, 100 µL of the sample supernatant was mixed with 200 µL of 0.4M Tris-HCl pH 8.9 and 10 µL of dithiobisnitrobenzoic acid (DTNB) 10 mM. After 5 min at room temperature, the absorbance was determined at 412 nm. The results are presented as nmol GSH/mg of protein compared to a GSH standard curve [58].

### 4.11. Histopathological Analysis

Kidney samples were collected and fixed in 10% buffered formaldehyde. Subsequently, the samples were embedded in paraffin, sectioned, and the tissue sections (5 µm) were stained with hematoxylin-eosin (HE), and examined under an optical microscope (Olympus OX31, Olympus, Japan) focusing on cortical regions. For histopathological evaluation, a score was adapted, as previously described [43]. The score considered the presence of hydropic degeneration (0—absent; 1—short (1–25%); 2—moderate (25–75%) and 3—abundant (>75%)), the presence of inflammatory cell infiltrate (0—absent; 1—short (<25 cells); 2—moderate (25–125 cells); 3—severe (>125 cells)), and the presence of necrosis, considering nuclear alterations/cytoplasmic alterations/loss of tissue arrangement (0—absent; 1—short (1–25%); 2—moderate (26–50%); 3—moderate/severe (51–75%) and 4—severe (>75%)). In addition, the characteristics of the cellular infiltrate (polymorphonuclear, mononuclear and mixed) were considered. The final score used the following scale: 0—no involvement; 1–3: short involvement; 4–6 moderate involvement, 7–10: severe involvement. Analyses were carried out by a blind evaluator. Results were expressed as mean value (variation) for each group of six animals.

### 4.12. Immunofluorescence Staining

Six hours after LPS stimulus, animals underwent a perfusion process using 4% paraformaldehyde (PFA) in phosphate-buffered saline (PBS) injected via the ascending aorta artery. Next, kidney was dissected and post-fixed in 4% PFA for 24 h. Kidney samples were incubated in saccharose 30% (*m*/*v*) (72 h, 4 °C) and embedded in optimum cutting temperature (OCT) reagent. The blocks were sectioned using cryostat equipment (CM1520, Leica Biosystems, Wetzlar, Germany) at 16 µm of thickness. In a prior phase of the technique, antigenic recovery was carried out through exposure to 90 °C followed by immediate cooling until 30 °C was reached. Then, all samples were blocked with 5% bovine serum albumin (BSA) in phosphate buffered saline with 0.3% Triton 100-X. The kidney was stained with mouse CD45 PE/Cy5 (1:500, cat. #103110, Biolegend [RRID: AB_312971], San Diego, CA, USA), as well as mouse Phospho-NFκB (1:200, cat. #sc-136548, Santa Cruz Biotechnology [RRID: AB_10610391], Dallas, TX, USA) and rabbit Kelch-like ECH-associated protein-1 (Keap-1) (1:200, cat. #8047, Cell Signaling Technology, Danvers, MA, USA), followed by goat anti-mouse Alexa Fluor 647 (1:500, cat. #A-21236, Thermo Fisher Scientific [RRID: AB_2535805], Waltham, MA, USA) and goat anti-rabbit Alexa Flour 488 (1:500, cat. # A-11008, Thermo Fisher Scientific [RRID: AB_143165], Waltham, MA, USA), respectively. The nuclei were stained with DAPI (1:500, cat. # 14285, Cayman Chemicals, Ann Arbor, MI, USA). Image acquisition and analysis of fluorescence intensity were performed in the cortex region of the kidneys using a confocal microscope (TSC SPB, Leica Microsystems, Manheim, Germany). Scale bars are presented in each representative image contained in the results section. Analyses were focused on cortical regions of samples and always performed by a blinded experimenter and measured using confocal microscope software to provide quantitative data for the experiments.

### 4.13. Statistical Analysis

We used G*Power software (version 3.1.9.7, Düsseldorf, Germany) to determine the sample size [61]. Statistical analyses were performed using GraphPad Prism statistical software (GraphPad Software, Inc., version 9.2, La Jolla, CA, USA). The first step was the analysis using the Shapiro–Wilk normality test and Brown-Forsythe homogeneity test to select parametric or non-parametric analysis. When Gaussian distribution and homogeneity were observed, the results were presented as means ± SEM (standard error of the mean) and analyses were made using one-way ANOVA followed by Tukey’s post-test. When non-Gaussian distribution and non-homogeneous data were observed, the results were presented as median and interquartile range (IQR), and data were analyzed using Kruskal-Wallis followed by Dunn’s test. There were six mice per group; the results were representative of two replicates, and differences were considered significant for *p* < 0.05.

## 5. Conclusions

We demonstrated that RvD5 reduces LPS endotoxemia-triggered acute kidney injury. RvD5 was shown to reduce biochemical, immune, molecular, and histopathological alterations in the kidneys of LPS endotoxemia in female mice. The mechanism of action of RvD5 was related to the inhibition of NFκB activation and reduction of Keap-1 that ultimately facilitated Nrf2 activity. Because of these molecular mechanisms, RvD5 reduced the endotoxemia-triggered production of cytokines and oxidative stress, thereby reducing the recruitment of leukocytes and kidney damage, reestablishing urea and creatinine to normal levels. Thus, RvD5 deserves consideration for further development as a treatment for endotoxemia-triggered inflammatory kidney injury. Future studies might investigate whether the activity of RvD5 is restricted to LPS endotoxemia or whether it could be used to treat kidney injury caused by other conditions such as toxic agents, auto-immune diseases, non-steroidal anti-inflammatory drugs, some antibiotics, severe burns, allergic reactions, and the breakdown of tumor cells.

## Data Availability

The data that support the findings of this study are available from the corresponding author upon reasonable request.

## Sample Availability

All compounds used in this study are available to buy from the sources indicated in the materials and methods section.
